# Decrypting structural and functional changes in LeuT_Aa_ at atomic level employing LRET

**DOI:** 10.1186/2050-6511-13-S1-A53

**Published:** 2012-09-17

**Authors:** Azmat Sohail, Simon Bulling, Peggy Stolt-Bergner, Oliver Kudlacek, Gerhard F Ecker, Michael Freissmuth, Thomas Stockner, Harald H Sitte, Walter Sandtner

**Affiliations:** 1 Institute of Pharmacology, Center for Physiology and Pharmacology, Medical University Vienna, 1090 Vienna, Austria; 2 Research Institute of Molecular Pathology, Campus Vienna Biocenter, 1030 Vienna, Austria; 3 Department of Medicinal Chemistry, University of Vienna, 1090 Vienna, Austria

## Background

Neurotransmitter:sodium symporters (NSS) are integral membrane proteins that mediate the reuptake of monoamine neurotransmitters previously released into the synaptic cleft. They are of pharmacological significance because they are the target of many clinically important drugs. LeuT*_Aa_*, a leucine/alanine transporter is a bacterial homolog to NSS. Crystal structures of LeuT*_Aa_* with open to outward, occluded and inward-facing states have already been resolved at high resolution. Hence, LeuT*_Aa_* serves as a good paradigm for exploring the structure-function relationship of NSS proteins.

## Methods and results

To investigate the structure-function relation in LeuT at atomic level we employ lanthanide-based resonance energy transfer (LRET). LRET-based measurements require the introduction of an LBT (lanthanide binding tag) to accommodate terbium as the donor element and fluorophores chemically linked to a cysteine residue as the acceptor element. LBT tags and cysteine are introduced at selected positions in LeuT*_Aa_*. Introduction of an LBT tag may lead to a functionally disturbed host protein. So to screen functional LBT-LeuT mutants we established the scintillation proximity assay in the lab. To date, after screening functional LBT mutants of LeuT*_Aa_*, we have measured the intramolecular distances at atomic level of LeuT in micelles. In order to validate these distances we want to see the distance changes after reconstitution into liposomes, a more native environment that allows to establish a sodium gradient; this is important since the NSS operates along a chemical gradient.

## Discussion

Our LRET measurements are expected to help us validate or propose models of substrate transport. Our future plan focusses on the reconstitution of LeuT*_Aa_* in liposomes to allow for distance measurements in a more native environment.

